# Gut Microbiota in Metabolic Syndrome: Differences in Microbial Signatures and Clinical Profiles

**DOI:** 10.3390/medicina62081435

**Published:** 2026-07-23

**Authors:** Giuseppe Guido Maria Scarlata, Andrej Belančić, Emidio Scarpellini, Almir Fajkić, Tomislav Meštrović, Roberto Vicinanza, Davor Štimac, Ludovico Abenavoli

**Affiliations:** 1 Department of Health Sciences, University “Magna Graecia”, 88100 Catanzaro, Italy; giuseppeguidomaria.scarlata@unicz.it; 2 Center for Chronic Liver Diseases, “Renato Dulbecco” University Hospital, 88100 Catanzaro, Italy; 3 Department of Basic and Clinical Pharmacology with Toxicology, Faculty of Medicine, University of Rijeka, Braće Branchetta 20, 51000 Rijeka, Croatia; andrej.belancic@uniri.hr; 4 Internal and Nutritional Unit, “Madonna del Soccorso” General Hospital, 63074 San Benedetto del Tronto, Italy; scarpidio@gmail.com; 5 Translational Research in Gastrointestinal Disorders (T.A.R.G.I.D.), Gasthuisberg University Hospital, KU Leuven, 3000 Leuven, Belgium; 6 Department of Pathophysiology, Faculty of Medicine, University of Sarajevo, 71000 Sarajevo, Bosnia and Herzegovina; almir.fajkic@mf.unsa.ba; 7 University Centre Varaždin, University North, 42000 Varaždin, Croatia; tmestrovic@unin.hr; 8 Institute for Health Metrics and Evaluation, University of Washington, Seattle, WA 98105, USA; 9 Leonard Davis School of Gerontology, University of Southern California, Los Angeles, CA 90007, USA; roberto.vicinanza@usc.edu; 10 Department of Gastroenterology, Clinical Hospital Centre Rijeka, 51000 Rijeka, Croatia; davor.stimac@uniri.hr

**Keywords:** obesity, type 2 diabetes mellitus, hypertension, dysbiosis, clinical profile

## Abstract

Metabolic syndrome (MetS) is a complex and heterogeneous condition characterized by the coexistence of obesity, type 2 diabetes mellitus (T2DM), hypertension, chronic low-grade inflammation, and metabolic dysfunction. Increasing evidence suggests that the gut microbiota plays a central role in the development and progression of MetS by influencing host metabolism, intestinal barrier integrity, immune activation, endocrine signaling, and vascular homeostasis. This narrative review summarizes current evidence regarding gut microbiota alterations across major obesity-related metabolic phenotypes, including obesity alone, obesity complicated by T2DM, and obesity associated with hypertension. Obesity is generally characterized by reduced microbial diversity, depletion of beneficial taxa such as *Faecalibacterium* *prausnitzii* and *Akkermansia muciniphila*, impaired short-chain fatty acid (SCFA) signaling, increased intestinal permeability, and metabolic endotoxemia. The coexistence of T2DM is associated with a more pronounced depletion of butyrate-producing bacteria, altered bile acid metabolism, impaired incretin signaling, and enhanced inflammatory activation that may contribute to insulin resistance and hyperglycemia. In hypertensive obesity, gut dysbiosis appears to preferentially involve disturbances within the gut–vascular axis, including reduced SCFA-producing taxa, increased trimethylamine N-oxide production, endothelial dysfunction, oxidative stress, and vascular inflammation. Although microbial signatures partially overlap among metabolic phenotypes, functional alterations in microbial metabolites and host–microbiota interactions may better explain disease heterogeneity than isolated taxonomic changes. Current evidence supports the potential role of microbiota-targeted interventions and integrated multi-omics approaches in future precision medicine strategies for cardiometabolic disease prevention and management.

## 1. Introduction

Metabolic syndrome (MetS) has emerged as one of the defining cardiometabolic challenges of the present century, driven by the global convergence of obesity, type 2 diabetes mellitus (T2DM), hypertension, dyslipidemia and chronic low-grade inflammation. Its rapidly increasing prevalence reflects profound changes in dietary patterns, physical activity, urbanization and aging populations. It is accompanied by a substantial rise in cardiovascular disease, chronic kidney disease, metabolic dysfunction-associated steatotic liver disease and premature mortality [1,2,3]. Although excess visceral adiposity remains the central biological substrate of MetS, clinical progression is remarkably heterogeneous. Some individuals with obesity maintain relative metabolic stability for prolonged periods, whereas others rapidly develop insulin resistance, hyperglycemia, endothelial dysfunction, vascular injury and multisystem metabolic disease [4,5]. This variability suggests that visceral adiposity alone cannot fully explain the transition from uncomplicated obesity to complex cardiometabolic phenotypes. In recent years, the gut microbiota has become increasingly recognized as a critical regulator of metabolic homeostasis and a potential integrative link between environmental exposures, host metabolism, immune activation, and cardiometabolic dysfunction. Beyond its taxonomic composition, the gut microbiota functions as a dynamic metabolic organ involved in nutrient processing, energy harvest, epithelial barrier maintenance, bile acid transformation, immune modulation, and endocrine signaling [6,7]. Experimental and clinical evidence indicates that obesity-associated dysbiosis is characterized not only by altered microbial diversity and compositional shifts, but also by functional disturbances involving short-chain fatty acid (SCFA) metabolism, intestinal permeability, microbial metabolite production, and inflammatory signaling. Through mechanisms linked to metabolic endotoxemia, oxidative stress, adipose tissue inflammation, and impaired insulin signaling, these microbial alterations may amplify the metabolic consequences of excess adiposity and contribute to disease progression [8,9,10,11]. Importantly, MetS should not be viewed as a microbiologically uniform condition. Emerging evidence supports the concept of phenotype-specific microbial signatures, whereby different cardiometabolic manifestations are associated with partially overlapping yet functionally distinct patterns of dysbiosis. In obesity complicated by T2DM, microbial alterations appear to preferentially involve the depletion of butyrate-producing bacteria, the disruption of glucose metabolic pathways, altered bile acid signaling, and the impairment of the incretin axis. By contrast, obesity associated with hypertension may be characterized by disturbances within the gut–vascular axis, including reduced SCFA-producing taxa, increased trimethylamine N-oxide (TMAO) production, endothelial dysfunction, immune activation, and vascular oxidative stress. Taken together, these observations suggest that obesity provides a common but heterogeneous metabolic context in which microbiota-related alterations are frequently observed. It should not, however, be regarded as a uniform baseline dysbiotic state, because metabolically healthy obesity and variation in visceral adiposity, dietary exposure, medication use, host factors, and microbial ecology are associated with divergent metabolic trajectories [4,5,12,13,14]. The coexistence of T2DM or hypertension may be associated with additional, partly overlapping, functional alterations rather than with discrete microbial states [12,13,14].

Unlike previous reviews that primarily summarize individual mechanisms linking gut microbiota to obesity, T2DM, or hypertension, this narrative review adopts a phenotype-oriented perspective by comparing microbial alterations across the major obesity-related metabolic phenotypes within MetS. We emphasize how differences in microbial functionality, including SCFA metabolism, bile acid signaling, TMAO production, intestinal barrier integrity, and inflammatory pathways may contribute to the clinical heterogeneity of MetS. In doing so, the present narrative review highlights the transition from descriptive taxonomic changes toward an integrated functional framework with potential implications for precision medicine.

The aim of this narrative review is to critically examine gut microbiota alterations and associated clinical characteristics across major metabolic phenotypes within MetS, with particular emphasis on obesity alone, obesity complicated by T2DM, and obesity associated with hypertension. Through the integration of phenotype-specific microbial signatures with their functional and clinical consequences, we propose a conceptual framework that explains metabolic heterogeneity within MetS beyond isolated taxonomic descriptions.

## 2. Literature Search and Conceptual Framework

### 2.1. Narrative Review Methodology

This article was designed as a narrative review to integrate epidemiological, mechanistic, translational and clinical evidence regarding gut microbiota alterations across major metabolic phenotypes within MetS including obesity, T2DM, and hypertension.

A key concept to keep in mind is the multidisciplinary nature of this rapidly evolving field, which encompasses gastroenterology, endocrinology, cardiometabolic medicine, microbiology, immunology, vascular biology, metabolomics, and nutritional sciences. As such, the primary objective was to synthesize current concepts across diverse areas of investigation rather than perform quantitative evidence pooling or formal systematic evaluation. This approach was considered most appropriate for developing a comprehensive, hypothesis-driven overview of phenotype-specific microbial signatures, host–microbiota interactions, microbial metabolite signaling, and the mechanisms linking gut dysbiosis with metabolic heterogeneity in MetS. Furthermore, the narrative framework facilitated the integration of emerging evidence supporting the concept that functional microbial alterations may better explain cardiometabolic disease progression than isolated taxonomic differences.

### 2.2. Information Sources and Search Period

A targeted literature search was conducted using PubMed and Scopus. To maximize literature coverage and identify additional relevant studies, manual screening of reference lists from key review articles, systematic reviews, meta-analyses, consensus statements, and original investigations was also performed.

The search included publications from 1 January 2011 to 1 April 2026, corresponding to the period during which substantial advances were made in next-generation sequencing technologies, microbiome research, metabolomics, host–microbiota interactions, and precision medicine approaches to cardiometabolic disease. Seminal historical publications published before 2011 were included selectively when considered essential for contextualizing foundational concepts, including early descriptions of obesity-associated gut dysbiosis, SCFA biology, bile acid metabolism, intestinal permeability, metabolic endotoxemia, and experimental evidence supporting the gut–metabolic axis.

### 2.3. Search Strategy and Eligibility Criteria

Search terms combined controlled vocabulary and free-text keywords related to metabolic syndrome, obesity, gut microbiota, microbial metabolites, and cardiometabolic disease. Representative search terms included: “metabolic syndrome”, “obesity”, “type 2 diabetes mellitus”, “hypertension”, “gut microbiota”, “gut microbiome”, “gut dysbiosis”, “intestinal microbiota”, “microbial diversity”, “short-chain fatty acids”, “butyrate”, “acetate”, “propionate”, “*Akkermansia muciniphila*”, “*Faecalibacterium prausnitzii*”, “trimethylamine N-oxide”, “bile acids”, “intestinal permeability”, “metabolic endotoxemia”, “lipopolysaccharide”, “gut-vascular axis”, “gut-brain axis”, “insulin resistance”, “endothelial dysfunction”, “oxidative stress”, “inflammation”, “precision medicine”, “probiotics”, “prebiotics”, “synbiotics”, and “fecal microbiota transplantation”.

Titles and abstracts were initially screened for relevance, followed by full-text assessment of potentially eligible publications. Articles were considered eligible if they fulfilled one or more of the following criteria:

(i) Characterized gut microbial composition or functional alterations associated with MetS and its subcomponents including obesity, T2DM, and hypertension;
(ii) Investigated molecular, cellular, metabolic, or immunological mechanisms linking gut dysbiosis with cardiometabolic dysfunction;
(iii) Evaluated microbial metabolites, including SCFAs, TMAO, bile acids, lipopolysaccharide (LPS), or other microbiota-derived signaling pathways relevant to metabolic disease;
(iv) Examined microbiota-targeted therapeutic strategies, including dietary interventions, probiotics, prebiotics, synbiotics, postbiotics, fecal microbiota transplantation (FMT), or other approaches aimed at modulating gut microbial composition or function;
(v) Provided epidemiological, translational, mechanistic, or conceptual evidence necessary to contextualize phenotype-specific microbial signatures and their relationship with cardiometabolic disease progression.

Eligible study designs included in vitro investigations, preclinical studies, animal models, observational studies, cross-sectional analyses, prospective cohort studies, clinical trials, translational research, metabolomic and multi-omics investigations, systematic reviews, meta-analyses, and relevant consensus statements. Where available, greater emphasis was placed on systematic reviews, meta-analyses, randomized clinical trials, and large prospective cohort studies, while experimental investigations were primarily used to support mechanistic concepts. Publications not available in English and studies unrelated to gut microbiota, metabolic syndrome, obesity, T2DM, hypertension, or host–microbiota interactions were excluded.

The relevance and inclusion of references were determined by team consensus involving internal medicine specialists and gastroenterologists (D.Š., E.S. and L.A.), a clinical pharmacologist (A.B.), an expert in experimental pathophysiology (A.F.), two microbiologists (G.G.M.S., T.M.) and a clinician-scientist specializing in aging and metabolism (R.V.), with L.A. acting as arbiter in cases of disagreement.

## 3. Gut Microbiota in Obesity

### 3.1. Reduced Microbial Diversity and Richness

Obesity is increasingly recognized as a heterogeneous condition associated with changes in intestinal microbiota. Within the MetS spectrum, it may serve as a useful clinical reference phenotype, rather than a uniform baseline metabolic or dysbiotic state, because metabolically healthy obesity and variation in visceral adiposity, diet, medication use, host factors, and microbial ecology are associated with divergent metabolic trajectories [4,5,15]. The gut microbiota can heavily influence energy balance, the integrity of the intestinal barrier, immune activation, hormonal signaling, lipid metabolism, glucose homeostasis, and the progression from a simple adiposity to a metabolically unhealthy phenotype. This is particularly important because not all individuals with obesity develop the same metabolic trajectory; some remain relatively metabolically preserved, whereas others progress toward insulin resistance, dysglycemia, vascular dysfunction, or fully expressed MetS. One of the most frequently reported findings in obesity is reduced microbial diversity, although this association is not uniform across all populations [16,17]. Lower α-diversity and reduced microbial gene richness may indicate a less resilient ecosystem, with a weaker capacity to maintain metabolic homeostasis during dietary, inflammatory or environmental stress. In other words, microbial richness may reflect metabolic vulnerability rather than only taxonomic composition [18,19].

Imbalanced or altered microbiota can have an effect on energy metabolism for the host. In particular, the microbiota from patients living with obesity can obtain more calories from non-digestible carbohydrates than a normal microbiota. This potentially allows for increased calories for the host [20]. The above effects could be related to changes to the expression of fasting-induced adipose factor; changes in hormones that affect appetite and metabolism, such as ghrelin, leptin, insulin, glucagon like peptide-1 (GLP-1), peptide YY; and changes in SCFA production or their respective signaling [21,22]. These mechanisms should not be interpreted as isolated microbial effects. Rather, they are part of a broader host–microbe metabolic network in which diet, adipose tissue expansion, gut barrier function, immune activation, and endocrine signaling interact continuously. However, reduced microbial diversity should not be interpreted as a diagnostic biomarker of obesity because human studies are strongly influenced by diet, fiber intake, geography, physical activity, sequencing methods, medication use, host genetics, and individual microbiota variability [23]. Therefore, reduced diversity may be better understood as a biomarker of reduced ecological resilience and metabolic vulnerability, rather than as a specific microbial fingerprint of obesity. In obesity alone, this altered microbial resilience may represent an early biological state that precedes more disease-specific microbial signatures observed in obesity complicated by T2DM or hypertension.

### 3.2. Taxonomic Shifts in Obesity

Several taxonomic changes have been described in obesity, but many findings remain inconsistent. The Firmicutes-to-Bacteroidetes ratio was historically proposed as a microbial signature of obesity, based on the assumption that some Firmicutes members increase energy harvest from the diet [24,25]. However, later studies showed conflicting results, with some reporting an increased ratio, others decreased Firmicutes, and some showed no significant difference [16,26,27,28]. Therefore, this ratio should be presented as a debated historical marker rather than a reliable biomarker of obesity. This point is essential because phenotype-specific interpretation of the gut microbiota should move beyond broad phylum-level comparisons and focus more on bacterial functions, strain-level variability, metabolite production, and clinical context.

More clinically relevant information may come from specific taxa. Obesity is frequently associated with depletion of beneficial bacteria such as *Faecalibacterium prausnitzii*, *Akkermansia muciniphila*, *Bifidobacterium*, *Lactobacillus*, and other butyrate-producing or mucosa-associated taxa [29,30,31]. Specifically, *F. prausnitzii* contributes to butyrate production and anti-inflammatory signaling, while *A. muciniphila* participates in mucin turnover, epithelial barrier function, immune modulation, and host–microbe communication [32,33]. Nevertheless, *A. muciniphila* should not be presented as a simple “anti-obesity bacterium” because its effects are likely to depend on strain or preparation, baseline abundance, diet, host context, and mucus-barrier status. Human intervention evidence remains preliminary. A proof-of-concept study reported acceptable tolerability and favorable changes in selected metabolic markers [29], whereas a subsequent randomized trial found no significant overall between-group differences in body weight or HbA1c, although exploratory findings suggested that baseline A. muciniphila abundance may modify response [33]. Thus, *A. muciniphila* remains a promising investigational candidate rather than an established microbiota-based therapy.

From a functional perspective, these taxonomic changes may be grouped into several clinically meaningful patterns. The depletion of butyrate-producing taxa, including *Faecalibacterium* and related organisms, may weaken epithelial energy supply and anti-inflammatory signaling. In contrast, enrichment of facultative anaerobes and LPS-containing bacteria may increase inflammatory potential, especially when intestinal permeability is altered. This functional grouping is more informative than a simple listing of bacterial taxa because it links microbial composition with mechanisms relevant to obesity-related metabolic dysfunction [34].

At the same time, obesity may be associated with the enrichment of potentially pro-inflammatory taxa, including Enterobacteriaceae, *Dorea*, *Streptococcus*, *Clostridium*, *Collinsella*, *Enterobacter cloacae*, and *Collinsella aerofaciens*. Enterobacteriaceae are particularly relevant because they contain LPS, which may promote metabolic endotoxemia when epithelial barrier integrity is impaired [35]. The combination of beneficial taxa depletion and LPS-containing bacterial enrichment may weaken epithelial defense and facilitate toll-like receptor 4 (TLR4)-mediated inflammatory activation [36]. It should be noted that these taxonomic patterns do not have the specificity needed to distinguish obesity from its related comorbidities but may provide a supportive microbial environment to develop into insulin resistance, dysglycemia, endothelial dysfunction, or hypertension.

### 3.3. Functional and Metabolic Changes

The functional profile of the gut microbiota may be more important than isolated taxonomic shifts. Classical experimental studies showed that an obesity-associated microbiota can increase energy harvest and transfer adiposity-related traits to germ-free mice [19,37]. However, human obesity cannot be explained by microbial energy extraction alone. Current evidence suggests that obesity involves disruption of key microbial functions, including altered energy extraction, SCFA signaling, bile acid metabolism, epithelial barrier integrity, and inflammatory regulation [27,38]. The functional view is especially relevant to MetS, where several clinical phenotypes can have decreased diversity and dysbiosis, but may have different dominant microbial pathways. In obesity, energy extraction, inflammatory responses to adipose tissue, and barrier dysfunction could be the main pathways. If a patient has T2DM compared to obesity alone, the pathways would include alterations in glucose metabolism and bile acid signaling. However, in obesity with hypertension, vascular metabolites and immune–vascular pathways could be more prominent.

SCFAs—particularly acetate, propionate and butyrate—are central microbial metabolites produced by fermentation of dietary fibers. They regulate epithelial barrier function, immune tone, gut hormone secretion, hepatic metabolism, appetite signaling, adipose tissue activity, and insulin sensitivity through G protein-coupled receptors such as GPR41 and GPR43/FFAR2, as well as through epigenetic mechanisms such as histone deacetylase inhibition [39,40]. In obesity, it is more accurate to state that SCFA metabolism and signaling are altered, rather than simply reduced. Some studies report lower abundance of butyrate-producing bacteria, whereas others show higher fecal SCFA concentrations, probably reflecting differences in fiber intake, microbial fermentation, intestinal absorption, transit time, and host utilization [41,42,43]. Thus, fecal SCFA concentrations should not be interpreted as a direct measure of SCFA biological activity. A high fecal SCFA level may reflect increased production, reduced absorption, altered transit, or impaired host utilization, whereas low levels may reflect reduced fermentative capacity or low dietary fiber intake. This complexity is important when comparing microbial signatures across metabolic phenotypes.

Gut microbiota can also modify bile acids through deconjugation and dehydroxylation, producing bile acid pools that signal through farnesoid X receptor (FXR) and takeda G protein-coupled receptor 5 (TGR5) [44]. These pathways regulate glucose and lipid metabolism, hepatic lipid handling, energy expenditure, inflammation, and intestinal barrier function. Therefore, obesity-associated dysbiosis may contribute to hepatic steatosis, adipose tissue inflammation, dyslipidemia, and insulin resistance [45,46]. In obesity alone, altered bile acid signaling may represent an early metabolic interface between dysbiosis, hepatic lipid accumulation, adipose tissue dysfunction, and reduced metabolic flexibility. In contrast, when obesity progresses toward T2DM, bile acid signaling may become more directly linked to impaired glucose homeostasis, insulin resistance, and incretin-axis dysfunction. In this context, the microbiota should be viewed less as a primary cause of obesity and more as a biological amplifier of diet-, adiposity- and inflammation-driven metabolic dysfunction [34,47].

Overall, functional alterations in obesity-associated microbiota may be summarized as a shift from a resilient, fiber-fermenting, barrier-supporting ecosystem toward a metabolically less flexible microbial community. This community may be characterized by altered fermentation, impaired epithelial support, modified bile acid signaling, increased inflammatory potential, and reduced capacity to buffer dietary and metabolic stress.

### 3.4. Pathophysiological Mechanisms

A central pathophysiological mechanism linking obesity and gut imbalance is increased intestinal permeability. High-fat and low-fiber dietary patterns may impair mucus quality, reduce epithelial defense, and weaken tight junction integrity. Obesity-associated dysbiosis—characterized by depletion of *Faecalibacterium*, *Roseburia*, *A. muciniphila*, *Bifidobacterium*, and *Lactobacillus* and enrichment of Enterobacteriaceae—may reduce occludin, claudin, and zonulin-1 expression [30,48,49]. This may be aggravated by reduced host defense peptides, mucus thinning, and impaired SCFA production or signaling [20,50]. Human studies have shown jejunal tight junction impairment in obesity, while lipid-induced permeability correlates with intestinal and systemic inflammation and may predict T2DM [50]. These observations support the concept that intestinal barrier dysfunction may be one of the earliest interfaces through which obesity-associated dysbiosis becomes systemically relevant.

The translocation of bacterial products, especially LPS, creates metabolic endotoxemia, defined as chronic low-grade elevation of circulating bacterial endotoxin, distinct from acute sepsis [33,51]. LPS binds LPS-binding protein and activates Cluster of Differentiation 14 (CD14)/TLR4 signaling, leading to nuclear factor-kappa B (NF-κB) activation and increased production of interleukin (IL)-6, IL-1β, tumor necrosis factor-alpha (TNF-α), and monocyte chemoattractant protein (MCP-1) [52,53]. This inflammatory signaling impairs insulin pathways in adipose tissue, liver, and skeletal muscle [19]. At the cellular level, inflammatory mediators may interfere with insulin signaling through serine phosphorylation of insulin receptor substrate proteins, activation of stress kinases, mitochondrial dysfunction, and oxidative stress. In adipose tissue, this may contribute to macrophage infiltration, adipokine imbalance, impaired lipid storage, and increased release of free fatty acids, thereby amplifying systemic metabolic dysfunction.

Overall, obesity-related dysbiosis creates a self-reinforcing loop: altered diet and adiposity reshape microbial ecology; dysbiosis weakens barrier function; LPS translocation activates inflammation; and inflammation worsens insulin resistance, hepatic lipid accumulation, adipose tissue dysfunction, oxidative stress, and metabolic inflexibility [54,55,56]. This relationship is unlikely to be strictly linear. Rather, obesity, diet, adipose tissue inflammation, intestinal barrier dysfunction, and microbial dysbiosis form a bidirectional network in which each component can reinforce the others. The gut microbiota should therefore be understood not as an isolated cause of obesity, but as a modifiable biological mediator that helps explain why obesity progresses toward MetS in some individuals [22,57,58].

## 4. Gut Microbiota in Obesity with Type 2 Diabetes Mellitus

### 4.1. Alterations in Gut Microbial Composition and Diversity

Obesity and T2DM are both associated with alterations in gut microbiota, and their coexistence is characterized by reproducible changes in microbial composition and diversity across populations [59]. In particular, recent studies in obese individuals with T2DM consistently report shifts in the relative abundance of key bacterial taxa, together with reduced microbial diversity and altered representation of metabolically relevant microbial groups. At the compositional level, the analysis by Ahmad et al. provided direct evidence that individuals living with obesity and T2DM exhibit a significantly altered gut microbiota composition compared with healthy controls, with identifiable shifts at both phylum and genus levels. In particular, the authors reported a relative reduction in Bacteroidetes and Verrucomicrobia, alongside a predominance of Firmicutes, resulting in an altered Firmicutes/Bacteroidetes ratio. This pattern is frequently associated with metabolic dysfunction. In addition, lower levels of SCFA-producing bacteria, such as *Roseburia* and *Faecalibacterium*, were also observed, alongside an increase in potentially harmful taxa like Enterobacteriaceae, suggesting a more pro-inflammatory gut environment. Notably, the authors linked these compositional changes to dietary patterns, showing that high-fat, low-fiber intake correlated with the loss of fiber-fermenting bacteria and enrichment of bile-tolerant and opportunistic taxa, reinforcing the diet’s central role in gut dysbiosis in this population [60]. Likewise, Wang et al. confirmed and expanded these findings by showing that obesity and T2DM together are associated with more pronounced alterations in gut microbial composition than obesity alone. Their group had much lower α diversity, which means fewer types of microbes and less resilience. There was a consistent loss of helpful Firmicutes, especially butyrate-producing Ruminococcaceae and Lachnospiraceae. At the same time, there were more pro-inflammatory taxa, including Proteobacteria and *Escherichia-Shigella*. The authors also found more *Collinsella*, which is linked to poor gut barrier function and dyslipidemia, and less *Akkermansia*, which supports gut health. These changes show a loss of protection and an increase in microbes that may promote inflammation and metabolic imbalance [61]. Subsequently, Huang et al. provided a detailed characterization of microbial composition in metabolically altered individuals. They identified distinct microbial signatures linked to obese and diabetic phenotypes. The study found enrichment of bile-tolerant and sulfur-metabolizing bacteria—such as *Bilophila*—and some members of the Clostridia class with possible pro-inflammatory or bile-acid-modifying effects. At the same time, beneficial bacteria involved in metabolic homeostasis, including SCFA producers and mucin-associated commensals, were depleted. *Bilophila* expanded in bile-rich environments and was strongly associated with high-fat diets and increased taurine-conjugated bile acids, connecting dietary fat intake, bile acid metabolism, and microbial selection. The authors found these microbial signatures correlated with clinical metabolic parameters, suggesting these taxa may be biomarkers of gut dysbiosis as well as contributors to disease progression. Indeed, in obese individuals with T2DM, gut microbiota changes involve a loss of SCFA-producing and barrier-protective bacteria and an enrichment of bile-tolerant, pro-inflammatory, and metabolically disruptive taxa [62].

### 4.2. Functional Consequences of Gut Dysbiosis

From a functional perspective, a central alteration reported in patients with obesity and T2DM is disrupted carbohydrate metabolism, which may be partly linked to gut microbiota changes. Lower levels of SCFA-producing bacteria are associated with reduced butyrate availability, compromising epithelial barrier integrity, decreasing mucin synthesis, and impairing G-protein-coupled receptor signaling, including GPR41 and GPR43 [63]. These receptors regulate insulin sensitivity, energy expenditure, and glucose homeostasis. Indeed, reduced SCFA availability has been associated with metabolic dysfunction [64]. In parallel, gut dysbiosis has been associated with alterations in bile acid metabolism with significant modifications in the conversion of primary to secondary bile acids, leading to an imbalance in the bile acid pool. This event disrupts signaling through key metabolic receptors such as FXR and TGR5, which regulate glucose metabolism, lipid homeostasis, and energy expenditure [65]. In this context, the enrichment of bile-tolerant and bile-modifying bacteria, as observed by Huang et al., further supports the central role of bile acid–microbiota interactions in diabetic obesity. Indeed, impaired bile acid signaling has been associated with reduced insulin sensitivity and poorer glycemic control [62]. The incretin axis can also be observed as another key nexus linking gut microbiota and host metabolism. Both SCFAs and bile acids stimulate secretion of GLP-1 from enteroendocrine L-cells [66]. Thus, the reduced SCFA-producing bacteria and disturbed bile acid signaling in obese T2DM patients may be associated with lower GLP-1 secretion, potentially contributing to impaired insulin secretion and hyperglycemia. These findings support a potential mechanistic link between microbial imbalance and endocrine dysfunction in T2DM [67]. Beyond metabolic pathways, inflammation driven by gut microbiota is potentially central to disease advancement. Gut dysbiosis increases intestinal permeability, enabling LPS entry into systemic circulation, a state termed metabolic endotoxemia. This activates TLR4 and cascades involving NF-κB, producing pro-inflammatory cytokines like TNF-α and IL-6. These cytokines disrupt insulin signaling, promoting resistance [68]. Elevated permeability and endotoxin-induced inflammation are essential mechanisms connecting gut microbiota to obesity and T2DM [69]. Taken together, the evidence demonstrates that gut microbiota changes in subjects living with obesity and T2DM encompass a complex interaction among compositional shifts, metabolic pathway dysfunction, bile acid signaling disturbance, incretin regulation, and immune activation.

## 5. Gut Microbiota in Obesity with Hypertension

### 5.1. Microbial Composition in Hypertensive Obesity

The coexistence of obesity and hypertension is associated with distinct alterations in gut microbial composition, extending beyond the dysbiosis observed in obesity alone [70,71,72,73,74]. An increasing body of literature shows the existence of a “gut–vascular axis” in which intestinal microbial imbalance has been associated with blood pressure regulation through metabolic, inflammatory, neural and vascular pathways [73,74,75,76]. In hypertensive obesity specifically, gut microbiota profiles are frequently characterized by lower microbial diversity and a significant decrease in beneficial SCFA-producing bacteria, but also by substantial enrichment of pro-inflammatory taxa associated with endothelial dysfunction and vascular inflammation [77,78,79]. One of the most reproducible findings in hypertension-associated dysbiosis is the reduction in SCFA-producing bacteria, especially considering members of the Lachnospiraceae and Ruminococcaceae families, as well as genera such as *Faecalibacterium*, *Roseburia*, *Eubacterium*, *Akkermansia* and *Butyricicoccus* [70,77,78,80]. These organisms are important producers of butyrate, acetate and propionate through the fermentation of dietary fiber [77,81]. SCFAs exert multiple beneficial effects on host physiology, including maintenance of epithelial barrier integrity, immune response modulation, regulation of sympathetic nervous system activity, and also direct vascular effects mediated through G protein-coupled receptors, e.g., GPR41, GPR43 and Olfr78 [82,83,84]. This means that a lower abundance of SCFA-producing taxa may contribute to impaired vascular homeostasis and elevated blood pressure. Although SCFAs are widely regarded as beneficial microbial metabolites, their role in obesity appears to be multifaceted and not entirely straightforward [85,86]. Increased SCFA production in individuals with obesity may enhance energy extraction from the diet, thereby potentially contributing to excess caloric availability and further weight gain [87,88]. Nonetheless, hypertension-associated dysbiosis does not appear to be defined by a single microbial signature; as in obesity, findings vary according to diet, ethnicity, medication exposure, sequencing methods, as well as comorbid metabolic conditions [77,86,89,90]. Relatively recent studies also suggest that hypertension may be associated with altered gut barrier integrity and increased translocation of microbial products such as LPS, further promoting sympathetic nervous system activation [91,92]. Experimental animal models have demonstrated that transplantation of hypertensive gut microbiota can induce elevated blood pressure in normotensive recipients, supporting a potential causative role of gut dysbiosis in hypertension pathogenesis [93]. However, the extent and direction of such associations remain inconsistent across studies, especially when it comes to research on humans [94], which means there is a need for longitudinal investigations to better define microbial signatures linked to hypertensive obesity. In obesity complicated by hypertension, gut microbiota imbalance may be amplified by adipose tissue inflammation, high-fat and high-salt dietary patterns, insulin resistance and endothelial dysfunction [72,73,77]. Several studies have reported enrichment of potentially pro-inflammatory and endotoxin-producing taxa, including increased abundance of *Escherichia coli*, *Klebsiella pneumoniae*, *Enterobacter cloacae* and other representatives of the phylum Proteobacteria and family Enterobacteriaceae [91,95,96]. Increased relative abundance of *Prevotella copri* and certain *Clostridium* species has also been linked to enhanced inflammatory responses and metabolic dysfunction in obese and hypertensive individuals [89,96].

### 5.2. Gut Microbiota-Derived Metabolites

Beyond compositional alterations, obesity-associated hypertension is increasingly linked to disturbances in microbiota-derived metabolites that influence vascular tone, endothelial function, renal regulation and systemic inflammation [77,94,95,96,97,98]. SCFAs may influence differentiation and expansion of regulatory T cells (Tregs), thereby limiting chronic low-grade vascular inflammation frequently detected in obesity and hypertension [99]. Different research groups further suggest that SCFAs may affect autonomic cardiovascular regulation through modulation of the “gut–brain axis”, including sympathetic nervous system signaling and vagal activity, which has an additional, compounding influence on blood pressure homeostasis and vascular reactivity [100,101,102].

Another microbiota-derived metabolite of interest in obesity-associated hypertension is TMAO, which is generated after the gut microbial conversion of dietary choline, phosphatidylcholine, and L-carnitine to trimethylamine, followed by hepatic oxidation [103,104,105]. In human studies, higher circulating TMAO concentrations have been associated with hypertension, impaired vascular reactivity, atherosclerotic disease, and adverse cardiovascular outcomes, progressing towards vascular dysfunction [106,107,108]. It also influences butyrate availability, which is important, as experimental studies have shown that butyrate suppresses NF-κB-mediated inflammatory activation, reduces vascular reactive oxygen species (ROS) production, and attenuates angiotensin II-induced hypertension [91,98]. Still, these findings are predominantly observational and cannot establish causality because circulating TMAO is influenced by kidney function, dietary intake, metabolic comorbidity, medication use, and host hepatic metabolism.

By contrast, cellular and animal studies suggest that TMAO-related pathways may promote vascular oxidative stress, endothelial inflammatory signaling, altered responses to hypertensive stimuli, and vascular remodeling [107,109,110,111]. The causal relevance and magnitude of these mechanisms in human obesity-associated hypertension remain uncertain. Accordingly, TMAO should currently be viewed as a context-dependent candidate metabolite rather than as a disease-specific biomarker or validated therapeutic target [94,97,112]. Microbiota–bile acid interactions represent another biologically plausible, but incompletely validated, pathway in obesity-associated hypertension. Gut bacteria participate in bile acid deconjugation and transformation of primary into secondary bile acids, thereby modifying bile acid pools and signaling through receptors such as FXR and TGR5 [113,114,115,116,117].

Experimental studies suggest that FXR/TGR5-related signaling may influence renal sodium handling, vascular reactivity, and blood-pressure regulation in animal models [118,119,120,121]. However, direct human evidence that microbiota-driven disturbances in bile acid metabolism cause hypertension remains limited. In human studies, altered bile acid profiles should therefore be interpreted as cardiometabolic associations rather than as proven causal drivers of hypertension. Prospective studies combining standardized bile acid metabolomics, detailed dietary and medication assessment, kidney-function adjustment, and ambulatory blood-pressure measurements are needed to clarify temporal and causal relationships.

### 5.3. Immune and Inflammatory Mechanisms

Chronic low-grade inflammation is considered one of the pivotal mechanisms linking obesity-associated dysbiosis with hypertension and vascular dysfunction [91,122,123]. In obese individuals with hypertension, changes in gut microbial composition and weakened intestinal barrier integrity may enable the rise of metabolic endotoxemia. Specifically, this relates to the translocation of microbial products (LPS, peptidoglycans and bacterial metabolites) into systemic circulation occurs, which in turn promotes a persistent activation of innate and adaptive immune pathways and also potentially contributes to chronic vascular inflammation [81,124]. There are specific mechanisms involved in this chronic process. LPS that stems from Gram-negative bacterial agents interacts with TLR4 and CD14 expressed on immune cells, endothelial cells, adipocytes and vascular tissues, and this interaction triggers the downstream activation of the NF-κB, mitogen-activated protein kinase and inflammasome signaling pathways [125,126,127]. These processes can then stimulate production of pro-inflammatory cytokines including TNF-α, IL-6, IL-1β, and MCP-1, which are implicated in endothelial dysfunction and vascular remodeling [128]. In obesity-associated hypertension, adipose tissue itself additionally acts as an important immunologically active organ characterized by macrophage infiltration and altered adipokine secretion [123,129]. Recent work by Paiva et al. demonstrated that gut microbiota alterations in patients with chronic kidney disease were associated with increased oxidative stress biomarkers and the accumulation of uremic toxins [130]. It is known that gut dysbiosis may influence immune cell polarization and vascular inflammation through the modulation of T helper 17 (Th17) cells and Tregs [131]. Increased Th17 activity and elevated IL-17 production have been associated with endothelial dysfunction, enhanced vascular oxidative stress and sodium-sensitive hypertension, whereas reduced Treg activity may impair anti-inflammatory homeostasis [132,133]. Experimental work performed by Wilck et al. revealed that high salt intake can induce depletion of *Lactobacillus murinus*, promoting Th17-mediated inflammation and aggravating salt-sensitive hypertension [131]. Moreover, hypertensive stimuli such as angiotensin II and high-salt diets have the propensity to alter gut microbiota composition, promoting pro-inflammatory immune activation at the same time [134,135]. Oxidative stress pathways represent another major mechanism connecting gut dysbiosis with hypertension in obesity [136]. Chronic inflammatory activation enhances the development of ROS within endothelial cells of the vasculature as well as in immune cells, adipose tissue and renal tissues. This takes place due to mitochondrial dysfunction, the uncoupling of endothelial nitric oxide synthase, and the activation of nicotinamide adenine dinucleotide phosphate (NADPH) oxidases [137,138]. Excessive generation of ROS decreases nitric oxide bioavailability, impairs endothelial-dependent vasodilation, promotes vascular stiffness, and contributes to hypertension-associated vascular remodeling [136,137,138]. It can also create so-called epigenetic metabolic scars within adipose tissue, which perpetuates obesity beyond initial triggers [136]. In this context, Santisteban et al. and Gong et al. have shown that gut dysbiosis in hypertension can be linked to a higher expression of subunits of NADPH oxidase (most notably NOX2 and NOX4) and enhanced vascular superoxide production (with subsequent disruption of endothelial redox balance), which can be viewed as a direct mechanistic link between intestinal microbial imbalance and oxidative vascular injury [122,139].

## 6. Microbial Signatures and Clinical Profiles Across Metabolic Phenotypes

**Figure 1:**
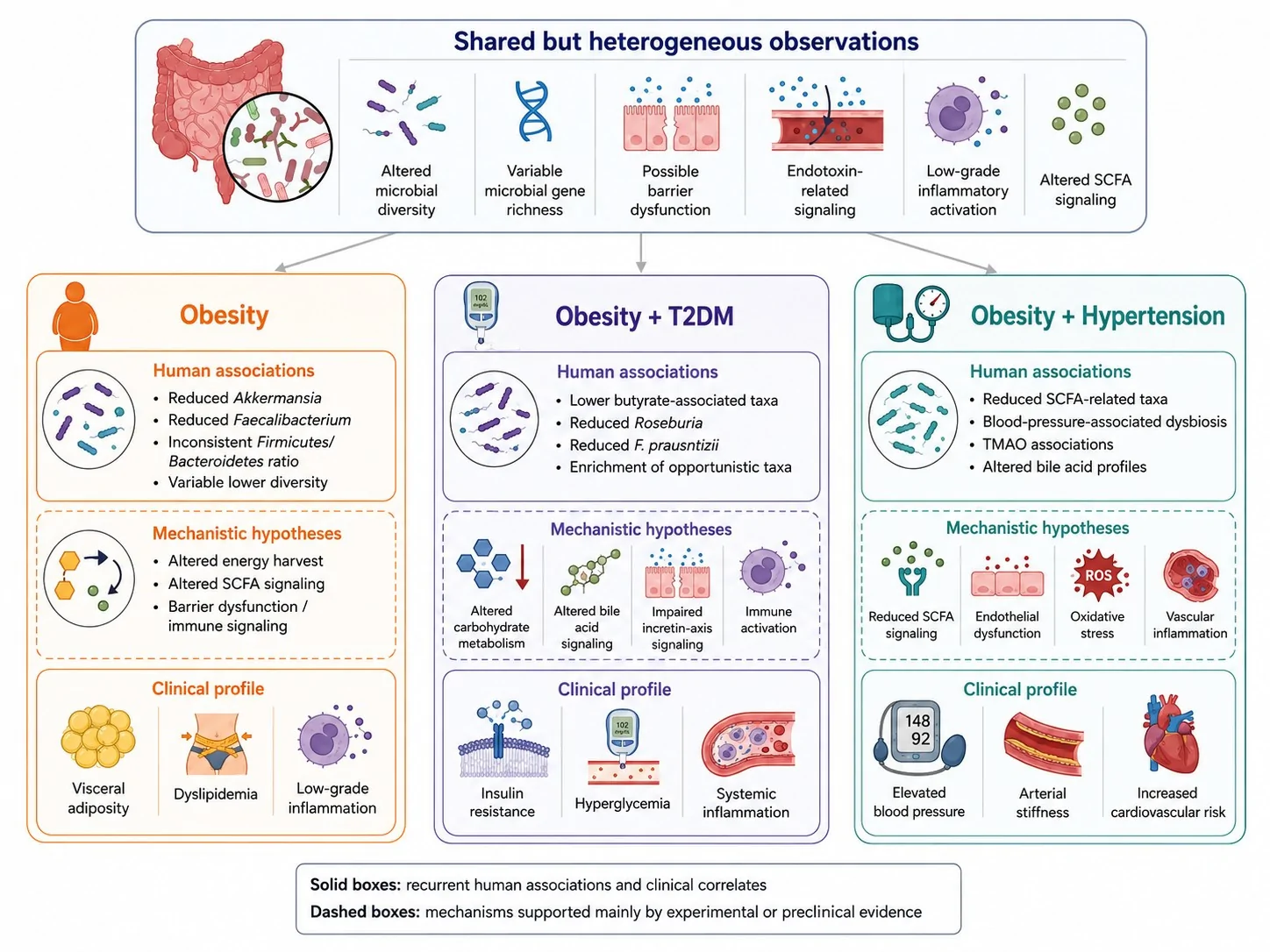
Shared and phenotype-enriched microbiota-related observations across obesity-associated metabolic phenotypes. Solid boxes indicate recurrent associations reported in human studies and clinical correlates. Dashed boxes indicate mechanisms supported predominantly by experimental or preclinical evidence. The figure does not imply diagnostic microbial signatures or establish causal relationships. Reported microbiota-related findings are influenced by dietary patterns, medication exposure, kidney function, obesity severity, geography, sequencing platforms, analytical methods, and coexisting disease. SCFA, short-chain fatty acid; LPS, lipopolysaccharide; T2DM, type 2 diabetes mellitus; ROS, reactive oxygen species; TMAO, trimethylamine N-oxide.

In summary, patients affected by obesity exhibit a gut microbiota imbalance. Figure 1 shows the gut microbiota signatures and clinical profiles across obesity-related metabolic phenotypes. This has been proposed to favor enhanced energy harvest from complex polysaccharides and may contribute to increased fat deposition [140]. Consistently, the depletion of mucin-degrading and barrier-protective bacterial taxa (e.g., *A. muciniphila*) has been correlated with higher body mass index (BMI). By contrast, preliminary human studies suggest that *A. muciniphila* supplementation may influence selected metabolic, inflammatory, and barrier-related measures in some individuals with overweight, obesity, or T2DM [29,33]. The available evidence, however, remains limited to small or heterogeneous study populations and formulations, and the most recent randomized data did not show a significant overall treatment effect on body weight or HbA1c. Any benefit may be restricted to subgroups defined by baseline A. muciniphila abundance [33]. Thus, this metabolic endotoxemia is mainly characterized by LPS, which, when translocated into the bloodstream, activates TLR4-NF-κB signaling. The latter has been shown in adipose macrophages and adipocytes, linking low-grade inflammation to the development of insulin resistance [141,142]. In addition, microbial metabolites, namely, SCFAs, can bind GPR41/43 to stimulate peptide YY and GLP-1 secretion. Clinically, this translates into appetite suppression and improved insulin sensitivity. Moreover, secondary bile acids produced by microbial bile salt hydrolase activity can activate FXR and TGR5, leading to inhibited lipogenesis and enhanced energy expenditure [143]. In addition, both animal models and clinical data show that prebiotic supplementation (e.g., inulin) can enrich microbial diversity and consistently increase SCFA production. These result in reduced body fat mass deposition [144].

T2DM is associated with reduced gut microbial diversity and favors a gut microbiota enriched in opportunistic pathogens, leading to the loss of butyrate-producing taxa (e.g., *Faecalibacterium prausnitzii*). Altogether, these alterations contribute to impaired barrier function and systemic inflammation [145]. Interestingly, gut dysbiosis decreases GLP-1 release. In fact, SCFAs and secondary bile acids physiologically trigger enteroendocrine L cells that secrete GLP-1. The latter leads to enhanced insulin secretion and improved glucose tolerance. Characteristically, T2DM subjects frequently exhibit hyperglycemia, which may be partially associated with alterations in the microbial-gut–pancreas axis [146]. *Lactobacillus rhamnosus* and dietary fiber supplementation have been shown to increase SCFA availability and may help modulate GLP-1 secretion. Preclinically and clinically, these steps translate into improved glycemic control [147]. Bariatric surgery can also perturb gut microbiota. In fact, patients undergoing Roux-en-Y gastric bypass exhibit a specific gut dysbiosis that enhances incretin responses and intestinal wall integrity. These correlate with remission of T2DM [79]. Fecal microbiota transplantation (FMT) and other microbiota-targeted interventions are being explored as strategies to modify dysbiosis and potentially influence metabolic dysfunction [148]. However, translational evidence from other disease settings indicates that microbiota modulation should not be viewed solely as a means of altering disease-related pathways. In hematological malignancies, gut microbial composition and function have been linked to drug metabolism, treatment response, gastrointestinal toxicity, and complications related to chemotherapy, immunotherapy, and hematopoietic stem-cell transplantation [149]. Although these observations cannot be directly extrapolated to MetS, they highlight that microbiota-targeted approaches may have both intended and unintended effects, shaped by concomitant medications, immune status, intestinal barrier integrity, and baseline microbial ecology.

A recent single-arm pilot study in six patients with alcohol-related cirrhosis reported that FMT was feasible and well tolerated, with no severe adverse events and preliminary improvements in selected clinical and quality-of-life measures, accompanied by changes in fecal microRNA profiles [150]. Nevertheless, the small sample size, absence of a control group, disease-specific setting, and short follow-up preclude conclusions regarding efficacy, durability, or generalizability to obesity, T2DM, hypertension, or MetS. Accordingly, FMT, live biotherapeutic products, and other microbiota-targeted interventions should currently be regarded as investigational in cardiometabolic disease. Future trials should incorporate standardized donor or product characterization; rigorous pathogen screening; systematic adverse-event surveillance; detailed assessment of diet and concomitant medication use; and clinically meaningful endpoints, including HbA1c, 24 h ambulatory blood pressure, visceral adiposity, liver fat content, medication requirements, and sustained cardiometabolic outcomes.

In patients with hypertension, gut dysbiosis is characterized by reduced microbial diversity, with an enrichment of mucin-degrading taxa (e.g., Muribaculaceae, Alistipes), and, conversely, a depletion of SCFA-producing genera (e.g., *Ruminococcus*, *Eubacterium eligens*). These dysbiosis features are associated with altered microbial metabolism: increased acetate-CoA ligase activity and decreased GPR43 signaling. They can contribute to elevated blood pressure by impairing the regulation of vascular inflammation and blood pressure dysregulation. In fact, FMT from hypertensive humans into germ-free mice has reproduced this mechanistic and clinical shift [151,152]. Not all obesity phenotypes carry the same cardiometabolic risk. Visceral adipose tissue appears to be more closely aligned than total adiposity or BMI with the major complications of obesity in the setting of metabolic syndrome [153]. Unlike subcutaneous fat, visceral fat is highly lipolytic, drains directly into the portal circulation, and promotes hepatic insulin resistance, atherogenic dyslipidemia, low-grade inflammation, and ectopic fat deposition in the liver, pancreas, skeletal muscle, and cardiovascular system [154]. Therefore, visceral adiposity represents a key biological link between obesity, T2DM, hypertension, and cardiovascular disease. This concept is clinically relevant because individuals with similar BMI values may have markedly different metabolic risk depending on visceral fat burden, waist circumference, and ectopic fat distribution [155]. Accordingly, the assessment of visceral adiposity may improve risk stratification beyond BMI and better identify patients with metabolically unhealthy obesity- and MetS-related complications.

Taxa differences seem to phenotipically distinguish obesity from other MetS features. By way of example, gut dysbiosis signatures seem more specific than those of obese patients suffering from T2DM and hypertension. In fact, diabetic and hypertensive patients share several taxonomic dysbiosis. Indeed, they have distinguished dysmetabolic pathways. We must note that there is also a significant overlap of metabolism pathways among obese and diabetic patients. Finally, the example of *A. muciniphila* highlights the issue of studies of heterogeneity on its dysbiosis in obesity. This paves the way for a better understanding of the extent of its dysbiosis and of its deranged metabolic behavior on the phenotype and metabolism, respectively, of patients suffering from obesity. World-wide studies with larger populations can fill the gap of data heterogeneity.

Therefore, we can consider it to be feasible and, in perspective, appealing, to use gut dysbiosis to distinguish phenotypic differences between obesity alone and T2DM and hypertension patients, considering them separately. Moreover, molecular biomarkers linked to differently altered metabolism can be useful to characterize T2DM and hypertensive patients. Altogether, this translates to a different therapeutic approach: obesity dysbiosis knowledge still appears limited and its modulation lacks solid data; T2DM patients can benefit from pre- and probiotics treatments, although data on FMT are still limited and lack consistency; hypertensive patients do not yet have solid evidence in favor of gut dysbiosis modulation by pre-, pro-, post- and synbiotics over FMT for their dysmetabolism modulation.

## 7. Limitations of Current Evidence

Despite the growing body of evidence linking gut microbiota to MetS, several methodological and biological limitations continue to hamper the interpretation and reproducibility of published findings. These limitations should be carefully considered when evaluating the reported associations between microbial alterations and obesity-related metabolic phenotypes. One of the major sources of heterogeneity is represented by environmental and host-related factors. Dietary habits, particularly fiber intake and overall dietary patterns, profoundly influence gut microbial composition and function, often exerting a greater effect than disease status itself. Likewise, geographical location, ethnicity, age, adiposity distribution, lifestyle, and host genetic background contribute substantially to interindividual variability, making comparisons across studies challenging and limiting the generalizability of reported microbial signatures. Recent international evidence has highlighted that geographical and dietary differences account for a considerable proportion of microbiome variability observed across human populations [156]. Medication exposure represents another critical confounding factor that is frequently insufficiently addressed. Metformin is well recognized to induce significant compositional and functional changes in the gut microbiota, including enrichment of *A. muciniphila* and SCFA-producing bacteria, making it difficult to distinguish disease-related dysbiosis from treatment-induced microbial changes. Similarly, GLP-1 receptor agonists appear to modify microbial communities through both direct metabolic effects and indirect consequences of weight loss and dietary changes. In addition, antibiotics, proton pump inhibitors, statins, and several other commonly prescribed medications may substantially alter microbial composition and metabolic activity and should therefore be systematically considered in microbiome studies [157,158,159]. Methodological heterogeneity further complicates the interpretation of the current evidence. Differences in stool collection and storage, DNA extraction procedures, sequencing platforms, targeted 16S rRNA regions or shotgun metagenomic sequencing, sequencing depth, taxonomic reference databases, and bioinformatic pipelines may generate substantial variability in microbial profiles independent of biological differences. Consequently, studies investigating similar clinical populations frequently report inconsistent taxonomic findings despite evaluating comparable phenotypes [160]. This variability helps explain why reproducible microbiota-based biomarkers remain elusive. Although several bacterial taxa, including *A. muciniphila* and *Faecalibacterium prausnitzii*, together with microbial metabolites such as SCFAs, TMAO, bile acids, and LPS, have repeatedly been associated with obesity, T2DM, and hypertension, none has yet demonstrated sufficient reproducibility, specificity, or external validation to support routine clinical application. Current evidence increasingly suggests that functional microbial characteristics, metabolic pathways, and host–microbiota interactions may provide more robust and biologically meaningful indicators than isolated taxonomic alterations.

## 8. Conclusions and Future Perspectives

MetS represents a biologically heterogeneous condition in which obesity, T2DM, and hypertension share interconnected metabolic and inflammatory mechanisms while exhibiting partially distinct gut microbial signatures. The evidence summarized in this narrative review indicates that obesity is frequently associated with alterations in microbial diversity, microbial metabolic functions, barrier-related pathways, and low-grade inflammation. These alterations are neither universal nor specific to obesity, and metabolically healthy obesity and substantial interindividual variation argue against defining obesity as a uniform baseline dysbiotic state. The addition of T2DM or hypertension appears to further reshape microbial composition and functionality toward more specialized metabolic phenotypes involving glucose dysregulation, altered bile acid metabolism, endothelial dysfunction, oxidative stress, vascular inflammation, and impaired host–microbiota signaling pathways. Importantly, current evidence indicates that the functional consequences of dysbiosis may be more clinically relevant than isolated taxonomic alterations. Disturbances in microbial metabolite production, including SCFAs, bile acid derivatives, and TMAO, together with increased intestinal permeability and metabolic endotoxemia, appear to represent central mechanisms linking gut microbiota to cardiometabolic dysfunction. These observations support the idea that gut dysbiosis should not be interpreted as a single disease-specific microbial fingerprint, but rather as a dynamic and context-dependent biological network influenced by diet, host genetics, adiposity, inflammation, medication exposure, and environmental factors. From a clinical perspective, further characterization of phenotype-specific microbial and metabolic alterations may improve our understanding of disease heterogeneity and could eventually contribute to future risk-stratification strategies. However, their clinical application in precision medicine remains speculative and requires validation in large prospective studies. Microbiota-targeted interventions, including dietary modulation, prebiotics, probiotics, synbiotics, postbiotics, and FMT, have shown promising preliminary results in improving metabolic and inflammatory parameters. However, current evidence remains limited by substantial inter-study heterogeneity, differences in sequencing methodologies, variability in dietary habits and pharmacological exposure, and the predominance of cross-sectional study designs. Moreover, the causal relationship between gut dysbiosis and the progression of metabolic disease has not yet been definitively established in humans. Future research should therefore move beyond descriptive taxonomic analyses toward integrated multi-omics approaches that combine metagenomics, metabolomics, transcriptomics, immune profiling, and clinical phenotyping. Longitudinal cohort studies and mechanistic interventional trials are particularly needed to clarify whether specific microbial pathways actively drive disease progression or simply reflect underlying metabolic disturbances. Greater attention should also be directed toward strain-level microbial characterization, functional microbial metabolites, gut barrier biology, and host–microbiota immune interactions. Ultimately, a deeper understanding of gut microbiota alterations across obesity-related metabolic phenotypes may help refine current pathophysiological models and inform the development of future microbiota-based interventions. Although the gut microbiota represents a promising source of biomarkers and therapeutic targets, its integration into precision medicine strategies will require robust longitudinal validation, mechanistic evidence, and standardized analytical approaches before clinical implementation. Current evidence, research gaps, candidate biomarkers, study priorities, and potential clinical applications of gut microbiota research across obesity-related metabolic phenotypes were summarized in Table 1.

## Figures and Tables

**Table 1:** Current evidence, research gaps, candidate biomarkers, study priorities, and potential clinical applications of gut microbiota research across obesity-related metabolic phenotypes.

Area	Current Knowledge Gap	Candidate Biomarkers	Priority Study Design	Clinically Relevant Endpoint
Microbial composition	Lack of reproducible microbial signatures across populations	*Akkermansia muciniphila*, *Faecalibacterium prausnitzii*, microbial diversity	Large multicenter longitudinal cohorts	Validation of phenotype-associated microbial profiles
Microbial function	Taxonomic changes poorly reflect biological activity	SCFAs, bile acids, TMAO, LPS	Integrated multi-omics studies	Identification of functional microbial pathways
Gut barrier integrity	Limited human evidence linking gut permeability with disease progression	Zonulin, LPS, LBP, intestinal permeability biomarkers	Prospective mechanistic studies	Prediction of metabolic deterioration
Host-immune response	Incomplete understanding of host–microbiota interactions	IL-6, TNF-α, CRP, immune-cell profiling	Translational human studies	Identification of inflammatory endotypes
Therapeutic interventions	Variable response to microbiota-directed therapies	Microbial metabolites, responder microbiome profiles	Randomized controlled trials	Improvement in insulin sensitivity, blood pressure, weight, HbA1c
Precision medicine	Lack of validated clinical algorithms integrating microbiome data	Combined microbiome + metabolome + clinical phenotype	Prospective validation cohorts	Risk stratification and personalized treatment

Abbreviations: CRP, C-reactive protein; HbA1c, glycated hemoglobin; IL-6, interleukin-6; LBP, lipopolysaccharide-binding protein; LPS, lipopolysaccharide; SCFAs, short-chain fatty acids; TMAO, trimethylamine N-oxide; TNF-α, tumor necrosis factor-alpha.

## Data Availability

No new data were created or analyzed in this study. Data sharing is not applicable to this article.

## Abbreviations

The following abbreviations are used in this manuscript:

BMI	Body Mass Index
CD14	Cluster of Differentiation 14
CRP	C-Reactive Protein
FMT	Fecal Microbiota Transplantation
FXR	Farnesoid X Receptor
GLP-1	Glucagon-Like Peptide-1
GPR41	G Protein-Coupled Receptor 41
GPR43	G Protein-Coupled Receptor 43
HbA1c	Glycated Hemoglobin
IL	Interleukin
LBP	Lipopolysaccharide-Binding Protein
LPS	Lipopolysaccharide
MCP-1	Monocyte Chemoattractant Protein-1
MetS	Metabolic Syndrome
NADPH	Nicotinamide Adenine Dinucleotide Phosphate
NF-κB	Nuclear Factor-kappa B
NOX	NADPH Oxidase
ROS	Reactive Oxygen Species
SCFAs	Short-Chain Fatty Acids
T2DM	Type 2 Diabetes Mellitus
TGR5	Takeda G Protein-Coupled Receptor 5
Th17	T Helper 17 Cells
TLR4	Toll-Like Receptor 4
TMAO	Trimethylamine N-oxide
TNF-α	Tumor Necrosis Factor-alpha
Tregs	Regulatory T Cells
